# Antiproliferative effect of the jararhagin toxin on B16F10 murine melanoma

**DOI:** 10.1186/1472-6882-14-446

**Published:** 2014-11-18

**Authors:** Durvanei Augusto Maria, Manuela Garcia Laveli da Silva, Mario Cesar Correia, Itamar Romano Garcia Ruiz

**Affiliations:** Biochemistry and Biophysics Laboratory, Butantan Institute, Av. Vital Brasil 1500, CEP 05503-900 Sao Paulo, SP Brazil; Genetics Laboratory, Butantan Institute, Sao Paulo, SP Brazil

**Keywords:** B16F10 murine melanoma cells, Snake venom, 1,10-phenanthroline, ECD-disintegrin, Jararhagin, *Bothrops jararaca*

## Abstract

**Background:**

Malignant melanoma is a less common but highly dangerous form of skin cancer; it starts in the melanocytes cells found in the outer layer of the skin. Jararhagin toxin, a metalloproteinase isolated from *Bothrops jararaca* snake venom acts upon several biological processes, as inflammation, pain, platelet aggregation, proliferation and apoptosis, though not yet approved for use, may one day be employed to treat tumors.

**Methods:**

B16F10 murine melanoma cells were treated with jararhagin (jara), a disintegrin-like metalloproteinase isolated from *Bothrops jararaca* snake venom, and jari (catalytic domain inactivated with 1,10-phenanthroline). Viability and adhesion cells were evaluated by MTT assay. The expression of caspase-3 active, phases of the cell cycle and apoptosis were assessed by flow cytometry. We analyze in vivo the effects of jararhagin on melanoma growth, apoptosis and metastasis.

**Results:**

The tumor cells acquired round shapes, lost cytoplasmic expansions, formed clusters in suspension and decreased viability. Jari was almost 20 times more potent toxin than jara based on IC_50_ values and on morphological changes of the cells, also observed by scanning electron microscopy. Flow cytometry analysis showed 48.3% decrease in the proliferation rate of cells and 47.2% increase in apoptosis (jara) and necrosis (jari), following 1.2 μM jara and 0.1 μM jari treatments. Caspase-3 activity was increased whereas G0/G1 cell cycle phase was on the decline. Proliferative rate was assessed by staining with 5,6-carboxyfluoresceindiacetate succinimidyl ester, showing a significant decrease in proliferation at all concentrations of both toxins.

**Conclusions:**

In vivo treatment of the toxins was observed reduction in the incidence of nodules, and metastasis and antiproliferative inhibition capacity. This data strengthens the potential use jararhagin as an anti-neoplastic drug.

## Background

Murine and human melanoma cells have been extensively used aiming at the evaluation of cellular and molecular changes induced by toxins from different snake venom metalloproteinases (SVMP). Effects on adhesion, migration and invasion of tumor cells through the extracellular matrix *in vitro,* and decreased number of metastasis *in vivo* were observed by a number of toxins, like eristostatin [1–3]; echistatin [4, 5]; contortrostatin [6–9]; salmosin [10–12]; *Vipera lebetina turanica* whole venom [13]; triflavin [14]; albolatrin [15]; and several others. Similar effects were demonstrated by oligopeptides whose design was based on particular sequences of the disintegrin domain of metalloproteinases [16, 17]. Jararhagin (jara), a multidomain toxin isolated from the venom of *Bothrops jararaca*, belongs to the PIII snake venom toxin family, and presents a catalytic Zn-dependent metalloproteinase domain, a disintegrin-like domain (ECD instead of RGD motif), and a cystein-rich domain [18]. Jararhagin stimulated migration and cytoskeleton rearrangement in normal epithelial cells, and recruited α_v_, but not α_2_ integrins [19]. It is known that jara binds to collagen and to the α_2_β_1_ integrin through two independent motifs, located on the disintegrin-like and cysteine-rich domains, respectively [20].

The chelating compound 1,10-phenanthroline inhibits Zn-dependent metalloproteinases, and was previously used to inactivate the catalytic domain of jara. Costa and Santos [19] showed inhibition on cells adhesion after treatment with jara inactivated with 1,10 orthophenantroline (jari). Accordingly, SK-Mel-28 human melanoma cells treated *in vitro* with jara or jari inhibited cells adhesion, besides inhibitory effects on morphology, proliferation and viability of cells. However, tumor cells migration and invasion were decreased *in vitro*, and a significant inhibition of tumor nodules multiplicity was observed mice genetically selected for acute inflammatory response [21]. Vitaxin (MEDI-522), a humanized antibody derived from the mouse LM609 monoclonal antibody, was recently reported to give positive results in a phase II trial enrolling patients with stage IV metastatic melanoma [22].

In the present study, the B16F10 cell line, a highly metastatic variant isolated from a spontaneous melanoma tumor from C57BL/6 J mouse [23], confirmed the previous study using SK-Mel-28 human melanoma cells. The effect of injection of B16F10 cells, pretreated with jara and jari, on allograph tumor growth and metastases *in vivo* was also investigated.

## Methods

### Cell cultures

The B16F10 murine melanoma cell line was obtained from American Type Culture Collection (*Mannasa, VA, USA*). The cells were maintained in 25–75 cm^3^ flasks containing RPMI-1640 medium (*Sigma Chemical Co., St. Louis, MO, USA*) supplemented with 10% heat-inactivated fetal bovine serum (*Cultilab, Campinas, BR*), 2 mM L-glutamine (*Sigma*), 100 U/ml penicillin and streptomycin (*Fontoura Wyeth AS*), in a humidified incubator at 5% CO_2_ and 37°C. For growth assays, cells were cultured in octuplicate using 96-wells flat bottom microplates (*Nunc, Int. Corp., Rochester USA*) at 2 × 10^4^ cells/well density. Cells were harvested at near confluence with 0.2% trypsin (*Sigma*) and counted for viability by trypan blue exclusion assay using the Malassez chamber.

### Isolation of jara and inactivation of its catalytic domain (jari)

Crude venom (20 mg) was fractionated in a FPLC phenyl-superose column, eluted, chromatographed on MonoQ column (FPLC), and the 52 kDa toxin band corresponding to jara was resolved on SDS-PAGE, and quantified by the Bradford method, as described [24]. Inactivation of the catalytic domain of jara was carried out through incubation with 10 mM 1,10-phenanthroline for 30 min at 37°C. The altered toxin was subsequently named jari.

### MTT assay

Viability of B16F10 cells was evaluated using the MTT [3-(4,5-dimethyl-thiazol-2-y1) 2,5-diphenyl tetrazolium bromide] (*Sigma Chemical Co., St. Louis, MO, USA*) colorimetric assay, that is based on the reduction of formazan crystals by living cells [25]. Briefly, B16F10 cells were seeded in 96-well tissue culture plates at 2 × 10^4^ cells per well and incubated for 24 h. The cells were treated with different concentrations of jara and jari (0.0005 to 1.2 μM); control cells were treated with PBS. Then, plates were incubated at 37°C under 5% CO_2_ for 24 h. After treatment, the supernatants were removed, 100 μL of 5 mg/mL MTT solution was added to each well, and the plate was incubated for 3 h. The precipitated formazan crystals were diluted in DMSO (*Sigma Chemical Co., St. Louis, MO, USA*) and measured at 540 nm using the microplate reader Thermo Plate (*Rayto Life and Analytical Science C. Ltd, Germany*). The IC_50_ value, which represents the concentration of toxin needed to decrease viability to 50%, as compared to untreated cells, was calculated from the concentration-response curve. Morphological changes induced on cells by jara and jari were observed through inverted light microscopy (*Carl Zeiss, Germany*), and the images were captured through CCD-IRIS, color video camera (*Sony Co.*), Cyto Viewer Lite Program.

### Cell adhesion assay

B16F10 cells were treated with jara (0.4 and 0.8 μM) and jari (0.2 and 0.4 μM) as described for the MTT assay. After 24 h incubation, the medium was removed and the cells were washed with 0.1 M PBS pH 7.2. Adherent cells were fixed with 0.1% glutaraldehyde, washed in PBS, stained with crystal violet 0.5% (*Sigma Chemical Co., St. Louis, MO, USA*), washed and then eluted with 100% ethanol. Absorbance was measured at 620 nm in ELISA counter (*Titertek Multiscan*) using the microplate reader Thermo Plate.

### Proliferative index by CFSE-DA

Methods used to perform proliferation analysis using CFSE-DA have been described previously [26]. Proliferation determination by CFSE-DA (5,6-carboxyfluoresceindiacetate succinimidyl ester) labelling method was adapted from a previously described protocol [27] that allows for direct detection of single proliferating cells, and facilitates quantification of cell division by flow cytometry, according to respective CFSE-dilution. Melanoma cells B16F10 treated with jara (0.4 and 0.8 μM) and jari (0.2 and 0.4 μM) for 24 h were analysed. Positive control group of lymph nodes of normal C57BL/6 J mice were cultured in RPMI-1640 medium supplemented with 2 mM L-glutamine (Cambrex, Belgium), 500 μM 2-mercaptoethanol (Sigma Aldrich, Germany), 20% heat-inactivated FCS and 1% penicillin/streptomycin (Cultilab, Brazil) at final concentration of 10^5^ cells/ml. Cell suspensions were cultured in U-bottom 96-well plates (Nunc, Denmark) with either 15 μg/mL phytohaemagglutinin (Sigma Aldrich, Germany) or medium alone. In each case a pellet of 10^5^ cells was resuspended in 1 ml of CFSE-DA labelling solution with concentrations ranging from 10 μM to 37 nM and incubated for 15 min at 37°C in the dark. After labelling, lymphocytes were incubated at 37°C in a 5% CO_2_ incubator for 96 h. Samples were transferred to flow cytometry tubes, and cells were counted using a FACSCalibur™ (BD – USA) flow cytometer. CFSE flow cytometric data files were analysed using Cell- QuestTM acquisition/analysis software (Becton Dickinson, San Jose, CA). Fifty thousand events were collected, and population proliferation was analysed using ModFitLT 2.0 software (Proliferation Wizard Methods).

### Scanning electron microscopy (SEM)

B16F10 cells (5 × 10^5^) treated with jara (0.4 and 0.8 μM) and jari (0.2 and 0.4 μM) for 24 h were washed with PBS and fixed in 2.5% glutaraldehyde pH = 7.2 at 4°C. Following 24 h fixation, the cells were rinsed in Na_3_PO_4_ buffer and were post fixed in 1% osmium tetroxide for 2 h at 4°C. Samples were dehydrated in increasing ethanol series, immersed in isoamyl acetate and at a critical point dried in liquid CO_2_ for 5 min. Samples were sputtered and coated with gold metal and examined in the *Leo 435VP Zeiss scanning electron microscope (Carl Zeiss, Germany).*

### Caspase-3 active in B16F10 melanoma cells

Caspase-3 active was determined by flow cytometry after treatment for 24 h with jara (0.4, 0.8, 1.2 μM), jari (0.1, 0.2, 0.4 μM), taxol 10 μM and untreated cells. Cells were washed with PBS and incubated with 1 μg caspase-3-specific antibody (*Santa Cruz, USA*) and with or without caspase-3 inhibitor (*Z-DEVD-FMK, Sigma*). Samples were incubated in a 5% CO_2_ incubator at 37°C for 1 h before flow cytometry quantification. All experiments were done through the FL2 channel by FACScalibur flow cytometer (*Beckton-Dickinson, San Jose, CA, USA*), with a minimum of 10,000 events acquired for each sample in three independent experiments.

### Apoptosis analysis by flow cytometry

B16F10 cells (2 × 10^5^) were seeded in 6-well culture plates and incubated at 37°C for 24 h. Cells were treated for 24 h with jara (0.4 and 0.8 μM) and jari (0.2 and 0.4 μM); control cells were treated with PBS alone. Cells found in the supernatant and adherent cells were incubated with specific binding buffer (10 mM Hepes, 140 mM NaCl, 2.5 mM CaCl_2_, pH = 7.4), containing 5 μL of annexin V-FITC (Santa Cruz, USA) and 1.8 ug/mL propidium iodide for 30 min at room temperature in the dark. After incubation, 400 μL binding buffer was added and cells were analyzed with FACScalibur (Becton DicKinson) using CellQuest software, determining the percentage of apoptotic cells.

### Cell cycle analysis

B16F10 cells were treated for 24 h with jara (0.4 and 0.8 μM) and jari (0.2 and 0.4 μM); control cells were treated with PBS alone. Supernatant and adherent cells were collected and washed in PBS. Cells and apoptotic bodies were harvested by centrifugation at 3000 rpm for 10 min. The cell pellet was fixed in ice-cold ethanol 70% and maintained overnight at −20°C. Prior analysis, PBS containing 1.8 μg/mL propidium iodide (*Sigma Chemical Co., St. Louis, MO, USA*) and 0.1 mg/L ribonuclease-A (*Sigma Chemical Co., St. Louis, MO, USA)* was added to the cell pellet and incubated in the dark for 20 min at room temperature. At least 10,000 events were acquired using CellQuest sotware. DNA content was measured in the FL2 channel on FACScalibur flow cytometer (*BD, USA*). The percentage of apoptotic cells and cells in the cell cycle phases G0/G1, S, G2/M and sub-haploid cells was determined using the ModFit 2.9 software.

### Experimental metastasis assays in vivo

B16F10 cells were treated with jara (0.8 μM) or jari (0.2 μM) during 24 h. Pretreated cells (5 × 10^4^) were injected subcutaneously in the dorsal region of each animal from three groups of 10 female C57BL/6 J mice. Untreated cells were injected in the control group. Mice were fed daily with ration *ad libitum* and water, and were killed 40 days later by administration of anesthetics, 160 μL of Ketamine hydrochloride was added to 400 μL of Xylazine, the prepared anesthetic agents were administered by intraperitoneal injects to mice at a volume 100 μL/g of body weight. The dorsal tumor developed at the site of injection was measured and the mean tumor volume was calculated [length (mm) × width^2^ (mm) × ¶/6)]. The ratio between tumor volumes of animals injected with untreated (control) versus toxin-treated MM cells was used to calculate toxin efficacy. Tumor tissues were removed and the number of metastatic lesions were quantified visually, measured, excised, fixed in 10% formalin and prepared for histopathology analysis. The experiments were carried out in accordance with the protocol of the Ethics Commission of Butantan Institute (CEUAIB N° 638/09).

### Cell cycle of lung metastasis cells

The metastases of lung were removed after necropsies proceed and perfused immediately with 1 ml of ice-cold enzyme solution [0.5 mg/ml deoxyribonuclease-I, 1 mg/ml collagenase type IV (Boehringer Mannheim) and 0.1 U/ml elastase (Sigma) in Tyrode’s buffer] for 30 min in a shaking water bath (250 rpm, 37°C). Cells released were separated from the tissue by filtration (100 μm mesh filters) and were pooled. After the cells had been washed twice in RPMI 1640 medium, the pellet was resuspended in fresh medium and cell counts were determined using the Malassez Chamber.

### Statistical analysis

Data were reported as mean ± standard deviation (SD), expressed as percentage or optical density of cell viability, adhesion *in vitro*; and number, incidence and mean volume of metastatic nodules. Statistical tests were performed by Student unpaired t-test and non-parametric Fisher’s-Yates exact tests (*GraphPad Prism software, version 5 for Windows, San Diego, CA, USA*). A probability of 0.05 or less was deemed statistically significant. The following notation was used throughout the text: *p < 0.05, **p < 0.01 and ***p < 0.001, relative to controls. The survival rates were calculated daily and the experiment was terminated when all the mice of control group died. Survival rate data were analyzed by Kaplan-Meier curves.

## Results

### Viability and adhesion effects of toxins treatment

**Figure 1:**
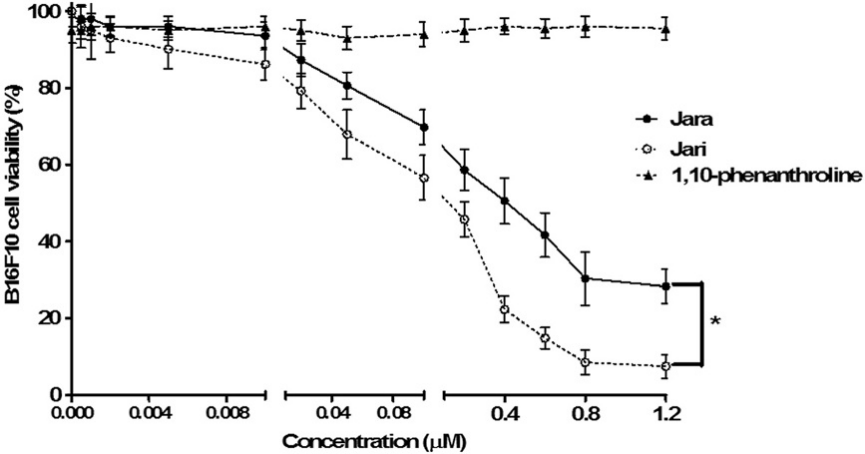
**Analysis of cell viability after treatment with toxins in B16F10 melanoma cell.** The viability of B16F10 melanoma cells was determined by the MTT assay after 24 h treatment using varying concentrations of jara, jari, and the chelating agent 1,10-phenanthroline. The viability curves regarding jara and jari treatments showed significant differences according to the Student unpaired t test (p < 0.05).

**Figure 2:**
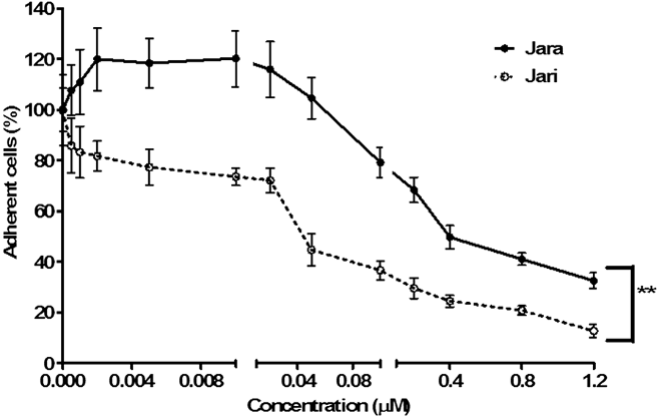
**Cell adhesion assay 24 hours after treatment with toxins in B16F10 melanoma cell.** The percentage of adherent cells after treatments was significantly different for jara and jari (p < 0.01) using by Student unpaired t test. The results are representative of three independent experiments.

The evaluation of jara and jari effects on B16F10 cell viability by the MTT assay also showed a dose-dependent cytotoxicity after 24 h. Concentrations equal or higher than 0.4 μM jara and 0.01 μM jari induced severe cytotoxicity that was statistically significant (*p < 0.05). According to the B16F10 viability curve, the IC_50_ values for jara and jari were 0.43 μM and 0.15 μM, respectively (Figure 1). No toxicity was observed in cells treated with 1,10-phenanthroline alone. In adhesion assays, concentrations higher than 0.1 μM jara or 0.01 μM jari significantly decreased the B16F10 cells adhesion in a dose-dependent manner (Figure 2).

### Determination of cell division rate following Jara and Jari treatments in melanoma cells B16F10

**Figure 3:**
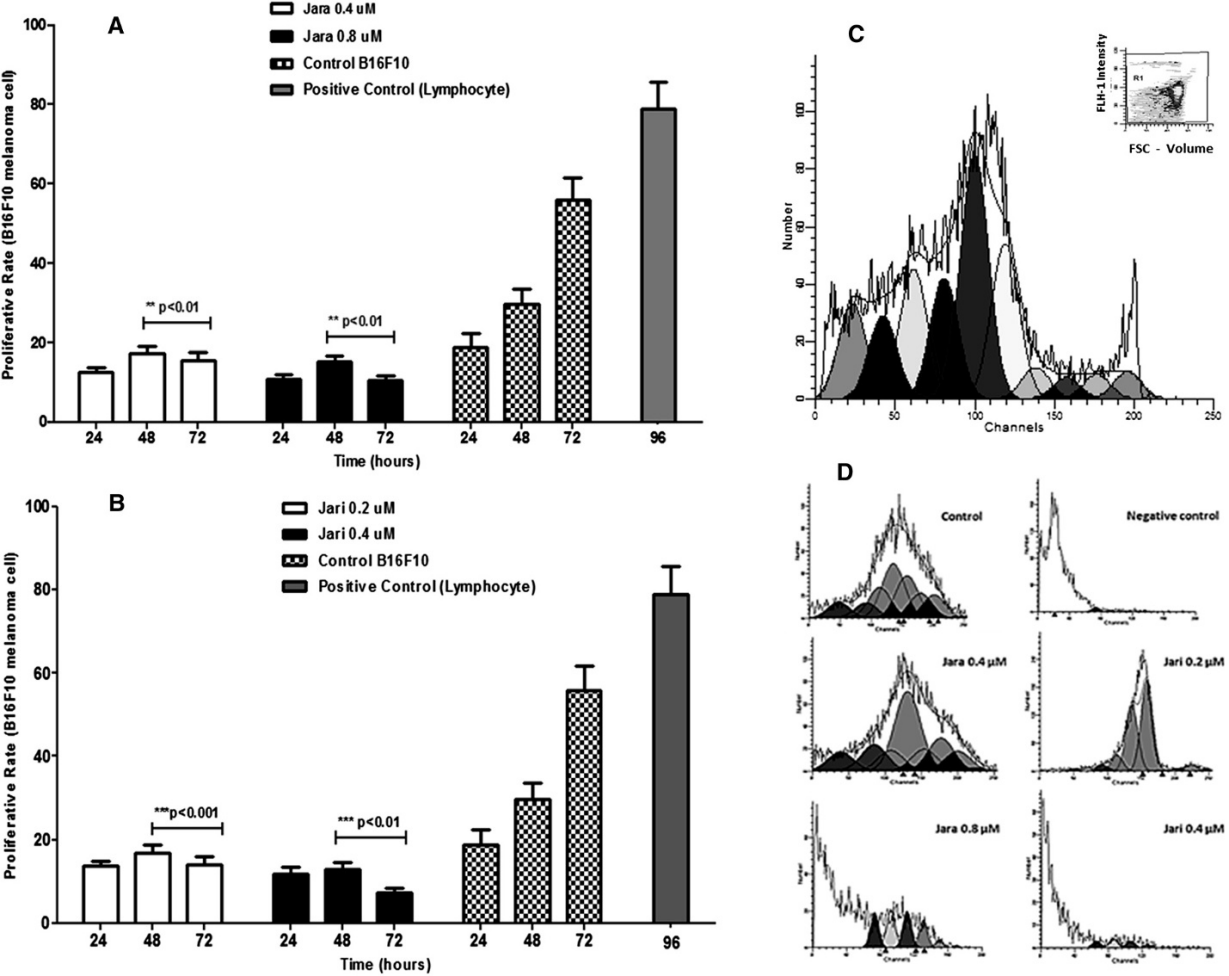
**Proliferative rate of B16F10 melanoma cells.** Toxins inhibit the proliferation and induce cell cycle arrest in B16F10 cells. Proliferation rates in B16F10 melanoma cells treated with jara and jari toxins for 24, 48 and 72 h determined using the ModFitLT 2.0 software. **(A)** The means proliferative rate using CFSE-DA assay of jara group; **(B)** CFSE-DA proliferation jari group; **(C)** CFSE-DA proliferation lymphocytes positive control (inset – dot plot representative); **(D)** Histograms represent the flow cytometric of CFSE-DA proliferative assay analyzes the proliferation of B16F10 melanoma cells after exposed to jara and jari toxins. All the experiments were repeated three times. Data are expressed as means ± SD. The statistical difference was obtained between control group non treated and toxins groups.

Melanoma cells were labelled with CFSE-DA to be used as control groups and were cultured for 24, 48 and 72 h; after culture, cells were harvested and analysed by flow cytometry. Cell division is characterized by sequential halving of CFSE fluorescence, generating equally spaced peaks on a logarithmic scale; peaks indicate the division cycle number. Similar results of MTT colorimetric assay were obtained using CSFE methodology, which accurately confirmed rate of proliferation of melanoma cells, in different periods of treatment with Jara and Jari. Figure 3 presents histograms acquired using ModFitLT 2.0 software and comparison of significant differences is shown in Figure 3A, B, C and D. Proliferation index showed a significant decrease in the population of melanoma cells at all concentrations of both toxins.

### Jara and Jari treatments induce morphological changes in B16F10 cells

Jara and jari were comparatively used in order to elucidate the role of the disintegrin domain of these toxins on the effects induced in tumor cells. The morphological effects on B16F10 cells were dose and time-dependent. These effects included loss of cytoplasm expansions, aggregates of round shaped cells, and detachment from the bottom of flasks, as shown by light microscopy similar to previous findings in SK-Mel-28 human MM cells [21, 28].

**Figure 4:**
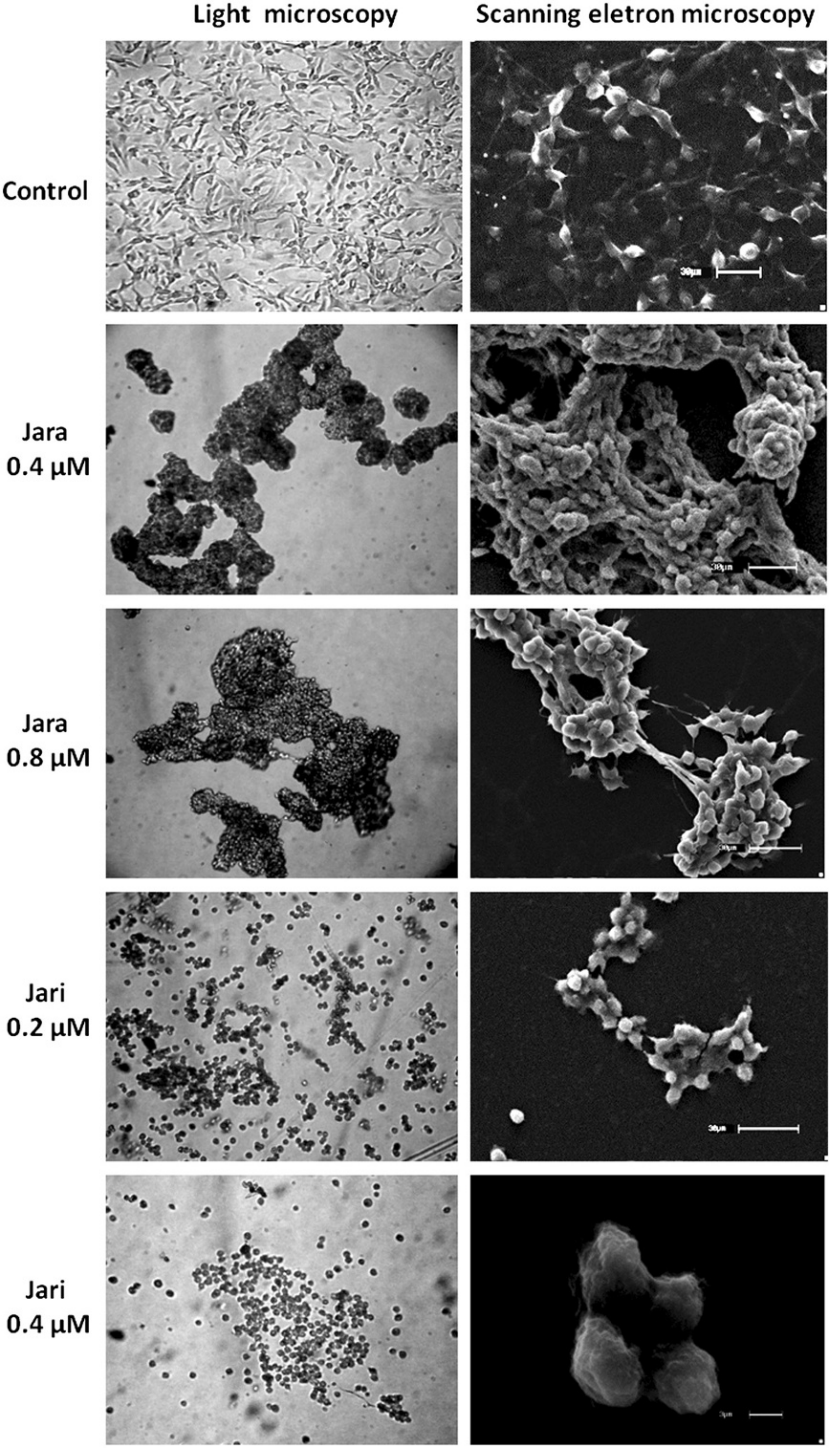
**Morphological features of B16F10 cells exposed to toxins.** The morphology of tumor cells was dramatically altered by toxin treatments, as observed by light microscopy. Scanning electron microscopy (SEM) showed the melanoma cells grown as a large, spreading monolayer, and the organization of the extracellular matrix with many protrusions. The cells showed retraction, and aggregates with 0.4 μM and 0.8 μM jara. Cells treated with jari showed detachment of the plate surface and formation of smaller aggregates (0.2 μM jari), and apoptotic bodies and necrosis debris (0.4 μM jari). Magnification 400× (microscopy), scale bar 30 μm and 3 μm (SEM).

Through SEM it was possible to reveal additional morphological details such as apoptotic and necrotic cells. Numerous membranes blebbings were induced by jara, whereas a decrease in cells density was observed with jari, which also changed the length and shape of the microvilli, caused shrinkage of cell volume, increased the number of apoptotic bodies and dispersed aggregates of the supernatant (Figure 4).

### Effect of toxins treatment on caspase-3 activity

**Figure 5:**
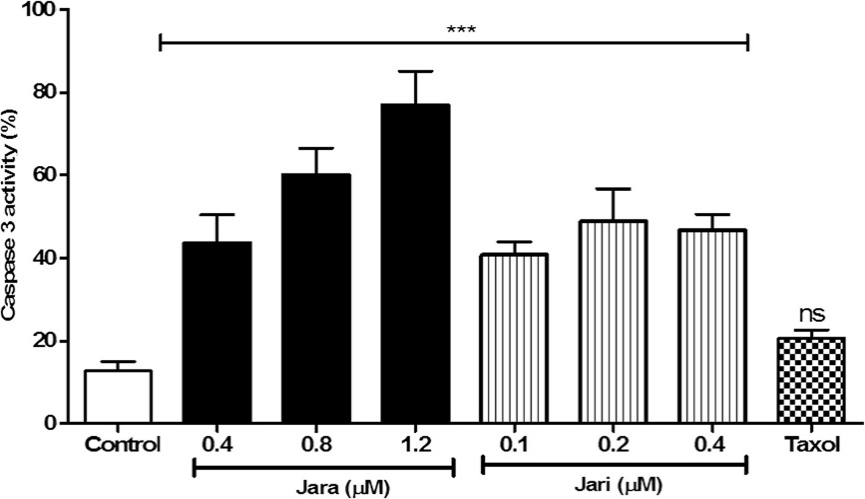
**Effects of toxins on caspase 3 active in B16F10 cells as evaluated by flow cytometry.** Treatments induced significant increase of caspase 3 active as compared to untreated cells or Taxol chemotherapeutic agent. Data were analyzed by the ANOVA one way variance test. The results are representative mean ± SD of three independent experiments, *p < 0.05.

Caspase-3 activation contributes to DNA fragmentation and morphological changes of cells. Flow cytometry analysis showed that jara and jari increased significantly caspase-3 activity in B16F10 cells. Treatments with 0.4, 0.8, and 1.2 μM jara induced caspase-3 activity on 43 ± 6.8%, 60 ± 6.5% and 77 ± 8.2% cells, respectively (***p < 0.001). In contrast, jari (0.1, 0.2 and 0.4 uM) increased the caspase-3 activity to about 45%, independently of concentration. Further treatments with 0.8 and 1.2 uM jari (not shown) killed most cells. Only 12.74 ± 2.2% untreated cells, and about 20 ± 1.9% cells treated with chemotherapy drug taxol (used as control) showed caspase-3 activity (Figure 5).

### Analysis of apoptosis and cell cycle by flow cytometry

**Figure 6:**
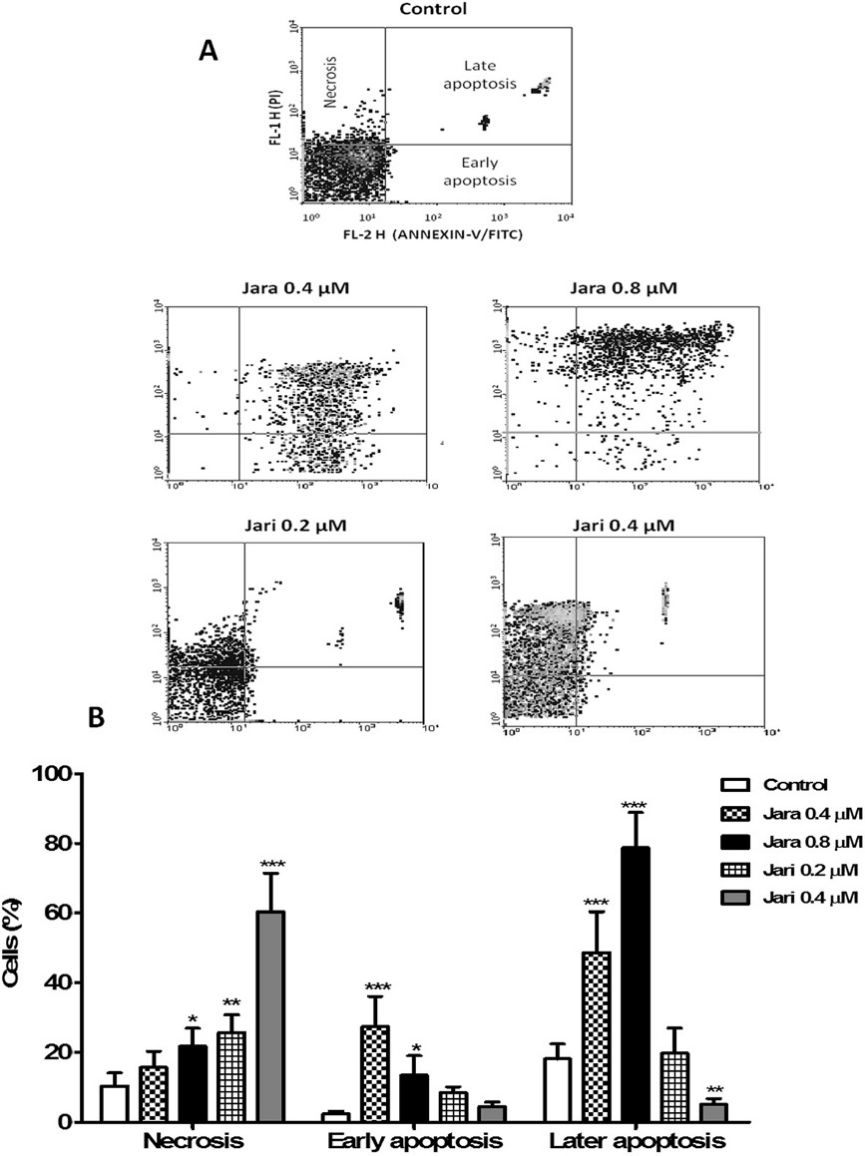
**Analysis of apoptosis by flow cytometry.** Induction of apoptosis in B16F10 cells by jara (0.4-0.8 μM) and jari (0.2-0.4 μM) for 24 h, as shown through Annexin-V/PI double staining by flow cytometry. The representative *dotplot* adquisitions showed apoptotic cells and necrosis areas **(A)**. The apoptosis rate was estatistically significant for the percentage of early/late apoptotic cells after jara treatment, while jari showed significant increase in the percentage of necrotic cells **(B)**. Data represent mean ± SD of three independent experiments. *p < 0.05 and ***p < 0.001.

Jara significantly increased early and late apoptosis of B16F10 cells. As shown in Figure 6A and B the number of jara treated cells in apoptosis was significantly increased (***p < 0.001) in a dose-dependent manner (0.4 and 0.8 μM), when compared to control cells. Contrarily, lower apoptosis and higher necrosis levels were observed (***p < 0.001) on jari treated cells (0.4 μM).

**Figure 7:**
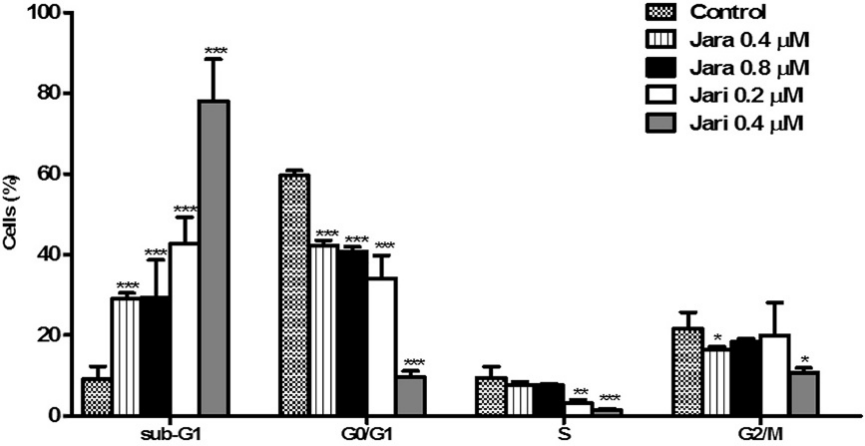
**Cell cycle analysis of B16F10 cells treated with jara and jari.** Cells were stained with iodide propidium for DNA content analysis by flow cytometry. The bars represent the proportions of G2/M proliferative cells; in phase S synthesis; G0/G1 quiescent cell, and debris in sub-G1. The figure shows a significant decrease in the percentage of cells in G0/G1 phase and a subsequent increase in sub-G1 phase with increased concentration of jara and jari. Data represents mean ± SD from three independent experiments. *Significantly different from control *p < 0.05; **p < 0.01 and ***p < 0.001.

The distribution of populations in the cell cycle phases was checked after jara and jari treatments. The percentage of cells in G_0_/G_1_ phases was significantly reduced by both toxins; the percentage of cells in S phase was significantly decreased only with 0.4 μM jari treatment. No significant alterations on the distribution of G_2_/M cells were induced by both toxins. Sub-G1 cell populations (debris and fragmented DNA) were significantly increased after jara and especially jari treatments (Figure 7).

### Inhibition of metastases in mice injected with toxin-treated B16F10 cells

**Figure 8:**
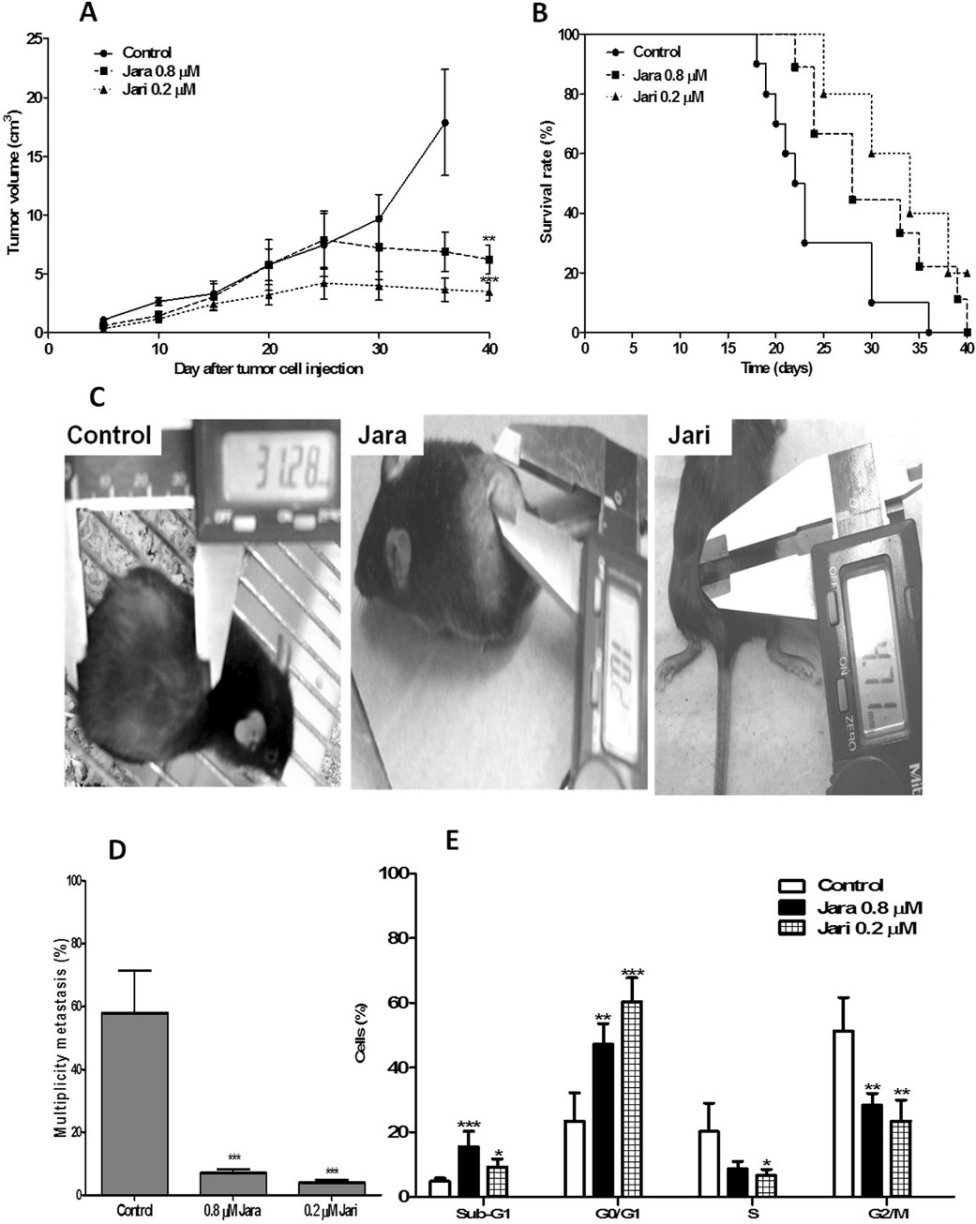
**Inhibition of tumor growth and metastasis dorsal treated with jara toxins and jari.** C57/Bl6J mice were administered via subcutaneous with B16F10 melanoma cells pretreated with 0.8 μM jara, 0.2 μM jari, and with untreated cells (control group). The growth curve **(A)** and macroscopic aspect of the melanoma dorsal tumor of the control and treated groups **(B)** are shown, as well as the Kaplan-Meier survival curve **(C)**, metastasis multiplicity **(D)** and cell cycle phase of lung metastasis **(E)**. Pre-treatment of tumor cells *in vitro* with jara or jari reduced the dorsal tumor volume of B16F10 melanoma cells, with statistically significant increase in survival rates with p < 0.05 (Log rank p < 0.018). The number of nodules in lung parenchyma was reduced on jara and jari groups. The distribution of cell cycle phase showed increase of tumor cells in sub-G1, fragmented DNA and decreased number of cells in proliferative response arrest in G2/M, caused by jara and jari on metastasis lung.

B16F10 cells treated with 0.8 μM jara and 0.2 μM jari were injected s.c. (5 × 10^4^ cells/mice) in C57BL/6 J mice. Untreated tumor cells were injected in the control group. Dorsal tumors were visualized in the injection site in all animal groups (100%) after the tenth day. The mean volume of dorsal tumors was significantly decreased in animals injected with cells pretreated with jara or jari (p = 0.0016) (Figure 8A). Dorsal tumor incidence was smaller in animals injected with cells pretreated with 0.8 μM jara (80%), but was 100% both in controls and the 0.2 μM jari group. After 40 days, the mean volume of dorsal tumors in controls was 17.8 ± 4.5 mm^3^ for control group; 5.8 ± 1.2 mm^3^ for 0.8 μM jara (p < 0.01), and 3.4 ± 0.7 mm^3^ for 0.2 μM Jari (p < 0.001), i.e., it was demonstrated a reduction in tumor volume of 67.5% (jara) and 86.5% (jari) compared with tumors developed by untreated B16F10 cells (Figure 8A, B). The survival probability (Kaplan-Meier curve) was significantly increased for animals injected with both jara and jari treated cells (log rank p = 0.00218), (Figure 8C). The long-rank test was used to test whether the difference between survival times between two groups: control X jara; control X jari and jara X jari is statistically different. Histopathological analysis of dorsal tumors induced by B16F10 cells pretreated with jara showed leukocytes infiltration, areas of regression-like fibrosis, and few blood vessels, as compared with controls (not shown).

The search for metastases was performed through macroscopic analyses of internal organs like lung, liver, spleen, kidney and lymph nodes. The number and volume of metastases decreased significantly in animals injected with jara and jari pre-treated cells (Table 1). The metastasis nodules multiplicity was also significantly decreased to 12% and 6.8% when the injected cells were pre-treated with jara or jari, respectively (Figure 8D).

**Table 1:** **Dorsal tumors and metastases induced in C57BL/6 after toxin treated B16-F10 murine melanoma cells**

	Control	0.8 μM JARA	0.2 μM JARI
Total n° mice	10	10	10
**Dorsal tumor**			
Incidence	10/10 (100%)	8/10 (80%)	10/10 (100%)
Mean volume (mm^3^)	17.8 ± 4.5	5.8 ± 1.2 (p = 0.01)	3.4 ± 0.7* (p = 0.0016)
**Metastasis**			
Nodules Incidence	10/10 (100%)	4/10 (40%)* (p = 0.0108)	2/10 (20%)* (p = 0.0007)
Mean volume (mm^3^)	42.10 ± 23.20	2.78 ± 6.10* (p < 0.0001)	1.31 ± 2.90* (p < 0.0001)
Multiplicity	5.8 ± 1.22	0.70 ± 1.05* (p < 0.0001)	0.40 0.96* (p = 0.0001)
**Metastases distribution**			
1- Lung	42 (72.4%)	02 (28.5%)	03 (75%)
2- Liver	01 (1.7%)	01 (14.2%)	0
3- Spleen	01 (1.7%)	01 (14.2%)	0
4- Kidney	05 (8.6%)	0	0
5- Lymph nodes	09 (15.5%)	03 (42.8%)	01 (25%)
**Total n° nodules**	58	07**p < 0.01	04***p < 0.001
		87.2%	93.1%

*The probabilities were compared to control values (Student unpaired T test; Fisher’s – Yates exact tests).

### Cell cycle of lung metastasis

Lung metastases induced by untreated and by jara and jari-treated cells were isolated, the cells were dispersed with collagenase type IV. A flow cytometric analysis regarding the DNA content of lung metastatic cells was performed. The analysis of the distribution of metastatic cells throughout the cell cycle phase suggested the induction of metastasis was differently affected by jara and jari pretreated tumor cells. The percentage of cells in G0/G1 and of AND fragmented cells (sub-G1) was increased, whereas an arrest of cells was found in S and G2/M phases (Figure 8E).

## Discussion

The search for anticancer agents has received a growing biomedical interest, in particular regarding the effect of native or isolated compounds found in snake venoms. The ability of jara to interfere with cancer mechanisms had already been investigated in SK-Mel-28 human MM cells revealing important cytological alterations, as detachment from the substratum of more than 80% cells, cytoplasm retraction and formation of cell clusters in suspension. The removal of the zinc ion from the metalloproteinase domain of jara by the chelating agent 1,10-phenanthroline caused a decrease in adhesion and viability of tumor cells compared with the effects promoted by native jara [21].

The present study using murine, instead of human melanoma cells, confirmed the above findings but at different toxin concentrations showing that human amelanotic SK-Mel-28 and the murine melanotic B16F10 cells have different sensibility to jara and jari treatments. Regarding the *in vivo* experiments, Corrêa Jr. et al. [21] worked with AIRmax and AIRmin mice, selected for high and low inflammatory response, whereas here C57BL/6 J mice were tested. C57BL/6 J mice originated in 1974, from the Jackson Laboratory, Bar Harbor, Maine, resistant development tumor spontaneous.

This study was improved by several new approaches, like the use of IC_50_ index for cells viability, and the inclusion of ortho-phenantroline group of treated dorsal tumor bearing mice. A clear distinction between increased apoptosis or necrosis was achieved by jara or jari treatments, respectively. It is worthwhile to point that Tanjoni et al. [29] demonstrated the induction of apoptosis (anoikis) in endothelial cells treated with jara. This fact indicates the caution as to the future use of jara or jari in the clinical field.

The cell cycle in eukaryotes controls progression, between and within the phases, through checkpoints that coordinate proliferation of the cells with the surrounding environment [30], and help ensure accuracy of DNA replication and division [31].

In order to decipher the suppressive mechanisms of jara and jari on B16F10 melanoma cells, we monitored the changes in cell cycle distribution by cytometry flow Toxin-treated cells showed increased sub-G1 populations, reduced G0/G1 and S, and arrest in the G2/M phases. In case of lung metastasis, most treated cells were arrest in G0/G1 and decreased G2/M cells, an arrest of untreated cells was observed.

Studies performed by ACTX-6, an L-amino acid oxidase from A. acutus showed flow cytometry analysis it could markedly increase accumulation of sub-G1 phase, which suggested induce apoptosis [32]. A basic polypeptide with 60 amino acid residue CTX-III from Naja naja atra venom exerts its specific anti-proliferative effects of hepatocellular carcinoma cell (HepG2) via S phase cell cycle arrest [32].

Measurement of cell proliferation with CFSE-DA offers many advantages over conventional 3H-thymidine incorporation assays. Labelling cells with CFSE-DA is a simple procedure that eliminates use of radioactive materials and results in an extremely bright fluorescent signal that is easily detected by table top flow cytometers. Intracellular esterase hydrolyses CFSE-DA into a fluorescent dye that binds covalently to cytoplasmic amino acid residues such as lysine [33]; thus, only viable cells are labelled. However, labelled cells that die during the culture period remain detectable until they disintegrate. Values obtained with the proliferative rate determined by CSFE-DA assay showed that effects on proliferative response of B16F10 melanoma cells when treated with Jara and Jari were similar to those results obtained by MTT assay. Lymphocytes were labelled with CFSE-DA to be used as control groups and were cultured for 96 h. After culture, the lymphocytes were harvested and analyzed. Cell division is characterized by sequential halving of CFSE fluorescence, generating equally spaced peaks on a logarithmic scale; peaks indicate the division cycle number. Similar results of MTT colorimetric assay were obtained using CSFE-DA methodology, which accurately confirmed proliferation rate of normal lymphocytes and cytotoxic effects to B16F10 melanoma cells, in different periods of treatment with jara and jari toxin.

The tumor proliferative rate decreases in proportion as it grows, increasing the doubling time. Thus, tumors have different doubling times at different times of their growth/stage. Macroscopically we observed the presence of areas of necrosis after 25 days of tumor growth, as well as, proportion of cells in necrosis. In all periods studied, there was an increase proliferative stage of cells at the G2/M in metastasis lung after 15 and 25 days of implantation.

The increased expression of the CASP3 gene, as measured by the caspase 3 protein fluorescence, confirmed previous data obtained by RT-PCR [28]. As a whole, those data point to a reduction of B16F10 cells proliferation attributed to jara and jari treatments. According to Baldo et al. [34], jara induces detachment and decrease the viability of human umbilical vein endothelial cells (HUVEC), at similar concentrations for SK-Mel-28 cells, while C2C12 myotube cells are more resistant to jara under the same conditions.

Most snake venom disintegrins containing a disintegrin-like/cystein-rich domain do not exhibit strong anti-proliferative activity. However, jara and jari showed anti-proliferative activity, besides decreased viability and detachment. SEM confirmed the ability of jara and jari to provoke detachment and apoptosis (jara) and necrose (jari) in B16F10 cells. Furthermore, inhibition of the catalytic domain of the native toxin improved the anti-tumor effects, suggesting the disintegrin-like/cystein-rich domains to be most important for the anti-proliferative effect. An alternative approach to this study was the pre-treatment of B16F10 cells with jara and jari in order to evaluate the toxin effect *in vitro* on the adhesion process. Again, it was observed that jari was more effective to inhibit adhesion than jara (not shown). These results reinforce the importance of the disintegrin-like domain of jararhagin for the adhesion inhibition, rather than the proteolytic activity of the catalytic domain. Accordingly, Costa and Santos [19] suggested the importance of the active catalytic domain of jara for normal cells migration, but not for adhesion. The apoptotic effect of jara and jari was further confirmed by the analysis of expression of caspase 3, the main apoptotic marker. Decrease in viability and adhesion of B16F10 cells was accompanied by enhanced expression of caspase-3. Once activated, caspase-3 targets specific substrates, such as actin and nuclear lamin A, and leads to DNA fragmentation, chromatin condensation, and formation of apoptotic bodies. This apoptotic effect of jara had already been observed on SK-Mel-28 human cells [21]; on tEnd murine endothelial [29] and HUVEC cells [34]. Contrarily to the effects induced by jara, and especially jari, on SK-Mel-28 and on B16F10 cells (this study), no morphological changes were detected when normal endothelial cells were treated with EDTA-inactivated jara [29]. The inactivation of the proteolytic domain of jararhagin would better fit the binding of the disintegrin/cystein rich domains to integrin receptors, improving signal transduction pathways [20]. These data suggest that the cytotoxic activity of jara and jari on the B16F10 cells are due to mechanisms other than direct cytolytic effect, but the exact mechanism is not yet clearly understood. Lipps [35] suggested that venoms act directly on tumor cells causing their lysis, whereas Markland et al. [36] proposed they act indirectly by destroying the microenvironment produced by the tumor cells. The present *in vitro* study shows that jara induces apoptosis and causes DNA fragmentation by activation of caspase-3, while jari induces necrosis and decreased proliferative response, in B16F10 melanoma cells.

Tumor metastasis is a dynamic process during which a number of complex interactions occur between tumor cells and the host. Metastasis causes the majority of morbidity and mortality associated with melanoma. The lungs are one of the most common sites of melanoma cell dissemination. Tumor progression and metastasis depends on factors that are intrinsic to tumor cells; on the extracellular matrix proteins organization; proteases; chemokines release; and cellular adhesion molecules [37]. Disintegrins are potent inhibitors of integrin-ligand interactions. SK-Mel-28 cells pre-treated with jara, and subsequently injected in mice selected for anti-inflammatory response significantly reduced the number of lung metastases [21]. In the present study, the decreased tumor volume, and the increased number of treated cells in G0/G1 and arrest in G2/M cell cycle phases observed in lung metastatic cells, can be considered important markers of reduced tumor burden and enhanced lifespan of mice bearing B16F10 cells. The incidence of metastasis was also significantly reduced in animals injected with B16F10 cells pretreated with 0.8 μM jara (12.1%) or 0.2 μM jari (6.9%), as compared with controls. Reduction in metastasis incidence had already been observed using SK-Mel-28 cells pretreated with 0.8 μM jara (42.8%) or 0.2 μM jari (30.7%), as compared with controls (83.3%), that had been injected in mice genetically selected for acute inflammatory response [38]. The main implication elicited from all studies is the antiproliferative properties of jara, attributed to its disintegrin domain, and the proliferation arrest *in vivo*. Assuming its medical relevance, it is important to get a better understanding of the mechanism of jara through the evaluation of gene expression profiles induced on tumor or normal cells after toxin binding and activation of integrin transduction signals. The reduction on incidence of nodules, the antiproliferative and antimetastatic effects induced by jara and jari strengthen the potential use of jararhagin as an anti-neoplastic drug.

## Conclusion

Inhibitory concentration IC50 obtained showed that Jara and Jari showed significant cytotoxicity in the tumor cell line B16F10 murine melanoma; Jari concentration of 0.4 mM was shown to be capable of inducing senescence population of cell cycle arrest leading to proliferation cell. Treatment with Jara and Jari toxins showed antiproliferative activity, decreased viability and adherence, and show that Jara induces apoptosis and causes DNA fragmentation through the activation of caspase-3, while Jari induces necrosis and decreased proliferative response in B16F10 melanoma cells. The values obtained with the proliferation rate determined by CSFE-DA assay showed that the effect on the proliferative response of B16F10 melanoma cells when treated with toxins corroborate the results obtained by MTT assay. In vivo treatment of the toxins was observed reduction in the incidence of nodules, and antimetastatic and antiproliferative effects in tumors. This data strengthens the potential use jararhagin as an anti-neoplastic drug.
